# Clinical Cases

**Published:** 1885-07

**Authors:** S. A. Kimball

**Affiliations:** Melrose, Mass.


### CLINICAL BUREAU.

---

### CLINICAL CASES.

S. A. Kimball, M. D., Melrose, Mass.

Case I. Mrs. K., thirty-seven years.

March 8th, 1885.—Grayish yellow membrane on both tonsils, began on left side; swallowing painful; breath very offensive; neck sore externally, worse on left side; cannot bear anything tight about throat; pains in back and all through head; very weak. Pulse 108. One dose Lach.$^{cm}$ (Swan).

P. M.—Throat more sore, worse on waking; tonsils more swollen; membrane not increased, looks softer; pain more in centre of throat on swallowing; thirsty; head and back feel better; cannot breathe through nose. Sac. lac.

March 9th.—Better; not as much pain on swallowing; no pains in back or head; membrane dirty yellow and softer looking; neck swollen more externally on left side; can breathe through nose; had a good night. Pulse 98. Sac. lac.

P. M.—Much better; less pain on swallowing; membrane coming off on right side. Pulse 92. Sac. lac.

March 10th.—Membrane about all gone, a little left on left tonsil; no pain on swallowing. Pulse 96. Sac. lac.

P. M.—Membrane all gone. Sac. lac.

March 11th.—Is up and about; throat all right. Sac. lac.

Examined urine March 15th, found no albumen.

Case II. Mr. A., thirty-eight years.

March 20th, 1885.—Greenish white membrane, on left tonsil; neck tender externally, left side; sharp pains to left ear on swallowing; pains all over; head sore; feels very weak. Breath very offensive. Pulse 96. One dose Lach.$^{cm}$ (Swan).

March 21st.—More membrane on left tonsil greenish white; great soreness of throat; does not like to sleep, as he can't collect himself for some time when he wakes; pains have left back and limbs and seem to be all in throat; membrane appearing on right tonsil; urine thick, dark, offensive. Pulse 96. One dose Lach.$^{cm}$ (Swan).

Gave this second dose because the disease was extending to the right tonsil. Perhaps it would have been as well to have waited.

March 21st, P. M.—Better; wakes all right; membrane looks softer and cheesy. Pulse 96. Sac. lac.

March 22d.—Membrane coming off, all gone on right and a large piece from left tonsil; has frequent sharp constricting pains in heart and left arm; much pain on swallowing; sleeps well. (He has had this pain in his heart before.) Sac. lac.

March 23d.—Only a small piece of membrane on left tonsil; less pain on swallowing. Sac. lac.

March 24th.—Throat all clear, feels all right. Sac. lac.

March 26th.—Constricting pains in heart as if grasped by a hand. (Has had this for some years.) Three doses night and morning Cactus grand $^{200th}$.

March 30th.—Found he had returned to work.

In Case I membrane began to come off in twenty-five hours.

In Case II in thirty-six hours.

